# What Is Known About Persons with Intellectual Disabilities and Cardiovascular Risk Factors—A Scoping Review

**DOI:** 10.3390/epidemiologia7030059

**Published:** 2026-04-25

**Authors:** Lisa Rein, Christine Tørris, Ana Carla Soares Portugal Schippert, Malin Holmström Rising, Astrid Torbjørnsen, Tina Rich Mogensen, Ann Kristin Bjørnnes

**Affiliations:** 1 Department of Nursing and Health Promotion, Faculty of Health Scinences, OsloMet—Oslo Metropolitan University, 0167 Oslo, Norway; christine.torris@oslomet.no (C.T.); anacarla@oslomet.no (A.C.S.P.S.); astridto@oslomet.no (A.T.); 2 Department of Nursing, Mid Sweden University, 85170 Sundsvall, Sweden; malin.holmstrom-rising@miun.se; 3 Department of Nursing and Nutrition, University College Copenhagen, 1799 Copenhagen, Denmark; tirm@kp.dk; 4 Department of Nursing and Health Sciences, Faculty of Health and Social Sciences, University of South-Eastern Norway, Campus Vestfold, 3184 Tønsberg, Norway; ann.k.bjornnes@usn.no

**Keywords:** cardiovascular risk factors, intellectual disability, metabolic syndrome, MetS, downs syndrome, Prader–Willi syndrome, obesity, overweight, waist, scoping review

## Abstract

Background/Objectives: Adults with intellectual disability are known to experience complex health needs, including an elevated presence of chronic conditions. Cardiovascular risk factors are a concern, yet the evidence base is fragmented, and the scope and focus of current research are not well understood. Methods: We conducted a scoping review to map the existing evidence on cardiovascular risk factors among adults with intellectual disability. The review included studies reporting on risk factor prevalence as well as participant characteristics (ethnicity, living arrangements, age, sex, and type of disability). Cardiovascular-related outcomes were extracted to clarify the health disparities documented in this population. Results: Searches of seven databases for studies published from 2013 onward yielded 15,598records, of which 85 met the inclusion criteria. Evidence was dominated by cross-sectional studies, with a few randomized controlled trials. Hypertension, Type 2 diabetes and obesity were commonly reported. Patterns appeared to reflect lifestyle, medication effects, genetic syndromes—particularly Down syndrome and Prader–Willi syndrome—and the severity of the disability. A notable share of the studies originated from the United Kingdom and the United States. Findings reveal a complex cardiovascular risk profile, emphasizing the need for tailored prevention and management. Conclusions: Adults with intellectual disability face a substantial burden of cardiovascular risk factors. Evidence on effective interventions remains limited, highlighting a need for targeted, evidence-informed approaches to improve cardiovascular health and long-term outcomes.

## 1. Introduction

Cardiovascular diseases (CVDs) are the leading cause of mortality globally, profoundly impacting public health [1]. CVD can partly be prevented by addressing modifiable risk factors such as hypertension, diabetes, obesity, physical inactivity, hyperlipidemia, smoking, and alcohol consumption [2]. To support this, both the American Heart Association and the European Society of Cardiology have developed comprehensive guidelines that emphasize early identification and management of these risk factors through evidence-based preventive strategies [3,4]. Accurate risk assessment and prediction are vital for identifying high-risk patients, ensuring they benefit most from preventive measures and lifestyle changes [5]. Prevention and treatment of CVD therefore remain high priorities internationally.

In the last decade, more evidence has emerged focusing on groups at high risk for CVDs, such as women [6]. Women experience higher rates of mortality, comorbidities, and complications, such as stroke, compared to men [7]. Concerningly, when disabilities are considered alongside general population characteristics, there is a notable lack of data despite the substantial risks involved, which may contribute to poorer health outcomes. In the United States, women with physical disabilities have significantly higher odds of (6.6 times greater) of developing CVD compared with women without disabilities. Furthermore, they are less likely to receive preventive care. Socio-economic disparities exacerbate the risk of increased disease burden and adverse cardiac events, as women with disabilities also tend to have lower levels of educational attainment, workforce participation, and annual personal income compared to the general population [8].

The American Association on Intellectual and Developmental Disabilities [9] defines intellectual disability as “limitations in both intellectual functioning and adaptive behavior.” Adaptive behavior includes conceptual skills (such as language, money, and time concepts), social skills (such as interpersonal relationships and problem-solving), and practical skills (such as daily living activities and work). The multifaceted nature of Intellectual Disability (ID) not only affects daily functioning but also significantly impacts overall health outcomes.

Individuals with ID experience poorer health than the general population and face a cascade of disparities, including CVDs, which negatively impact their overall health status and mortality rates [10]. Nearly 60% of deaths among individuals with ID are reported to be avoidable [11]. A recent retrospective cohort study of 3642 individuals [12], indicated that individuals with ID are at increased risk of CVD, particularly ischemic stroke, and have higher mortality from cardiovascular disorders. Similarly, another longitudinal study using data from 2009 to 2010 and follow-up from 2020 to 2023 [13] found that, compared with the general population, older adults (50+) with ID (N = 598) have a lower incidence of myocardial infarction and a similar or higher incidence of heart failure and stroke. High blood pressure, Down syndrome (DS), and antipsychotic use were key factors associated with increased cardiovascular risk

A higher proportion of individuals with ID live with obesity, due to genetic factors, medications associated with weight gain, and unhealthy lifestyle habits [14]. A previous systematic review and meta-analysis reported that more than one-fifth of individuals with ID have metabolic syndrome (MetS), a cluster of cardiovascular risk factors [15,16]. Yet, the prevalence of MetS and its associated risk factors is often recognized in specific populations [7,8], particularly among individuals with ID [17]. This group is frequently overlooked in public health discussions, even though individuals with ID may face a disproportionately high burden of cardiovascular risk factors [13,14,18].

The knowledge gap encompasses several areas. First, the prevalence of cardiovascular risk factors in individuals with ID is likely underestimated, as these factors are not systematically mapped or sufficiently acknowledged within this population [6,7,19]. Second, targeted research remains limited; individuals with ID are frequently excluded from epidemiological studies, resulting in an incomplete understanding of their specific health risks and needs [17]. Third, when disability status is considered within broader population health analyses, data remain sparse despite clear indications of elevated vulnerability. This lack of robust evidence may contribute to poorer outcomes and hampers the development of tailored public health strategies, ultimately limiting the efficient allocation of healthcare resources. Closing this knowledge gap is essential to reducing health disparities and improving the quality of life for this vulnerable population.

### Characteristics and Classification of Intellectual Disability

In this review, we refer to the concept of ID as outlined by the American Association on Intellectual and Developmental Disabilities (AAIDD). According to the AAIDD, ID is a condition that originates before the age of 22 and is characterized by significant limitations in intellectual functioning and adaptive behavior [19].

Intellectual functioning, also known as intelligence, refers to general mental capacity that involves learning, reasoning, and problem-solving. It is often measured by IQ tests, with a score of 70 to 75 indicating a significant limitation in intellectual functioning.

Adaptive behavior encompasses a range of conceptual, social, and practical skills that individuals perform in their everyday lives. Conceptual skills include language, literacy, and self-direction; social skills involve interpersonal skills, self-esteem, and social problem-solving; and practical skills relate to daily activities, occupational skills, healthcare, and safety. Limitations in adaptive behavior can also be determined through standardized tests [9,19,20].

The onset of ID occurs during the developmental period, i.e., before the age of 22. ID is one of several conditions known as developmental disabilities, such as DS and Prader–Willi Syndrome (PWS). DS is a genetic disorder caused by the presence of all or part of a third copy of chromosome 21. It is typically associated with physical growth delays, characteristic facial features, and mild to moderate ID. The average IQ of a young adult with DS is 50, equivalent to the mental ability of an 8 or 9-year-old child, but this can vary widely. PWS is a complex genetic condition affecting many parts of the body. In infancy, this condition is characterized by weak muscle tone (hypotonia), feeding difficulties, poor growth, and delayed development. Beginning in childhood, affected individuals develop an insatiable appetite, which leads to chronic overeating (hyperphagia) and obesity. Some individuals with PWS, particularly those with obesity, also develop type 2 diabetes mellitus in adulthood [9,19].

This scoping review aims to provide a comprehensive analysis of the prevalence of cardiovascular risk factors among individuals with ID, while also capturing data on ethnicity, living conditions, age/sex, type of disability, and outcomes related to cardiovascular risk to explore the specific health disparities faced by individuals with ID. By synthesizing existing research and presenting new data, this study seeks to advocate for increased awareness and proactive measures to mitigate these risks. Ultimately, the goal is to improve the health outcomes and quality of life for this often-marginalized group.

## 2. Materials and Methods

Initially, this study was planned as a systematic review, and the protocol was pre-registered on PROSPERO (#CRD42024485617) to ensure transparency and reproducibility. However, during the preliminary stages of the review, we identified a large and diverse body of studies. This diversity, spanning multiple areas of interest and including studies with varying designs and outcomes, made a traditional systematic review and meta-analysis impractical. As a result, we decided to change our approach towards a scoping review. Scoping reviews are particularly useful for mapping out broad and complex topics, providing an overview of the existing literature, irrespective of the study design [21]. This approach will enable us to comprehensively map the volume, nature, and characteristics of the primary research on the prevalence of cardiovascular risk factors in adults with ID. It will also help identify gaps in current research, informing future studies.

Scoping reviews, as established by Arksey & O’Malley [22], are designed to comprehensively map the literature on a particular topic, aiding in the identification of gaps in current research. In our review, we utilized their proposed five-stage framework, which includes: (1) formulating the research question, (2) identifying relevant studies, (3) selecting studies, (4) charting the data, and (5) collating, summarizing, and reporting the results.

### 2.1. Identifying the Research Question

This study aims to assess and summarize the existing evidence on the prevalence of cardiovascular risk factors in persons with intellectual disabilities.

### 2.2. Identifying Relevant Studies

Inclusion criteria for the study encompass research that involves individuals aged 18 and up with an ID and reports on the prevalence of cardiovascular risk factors. We focused on studies published in English or European languages after January 2013. The study designs considered for inclusion were quantitative—experimental and observational—including cohort and cross-sectional studies.

Studies with mixed groups of children and adults, or adults with and without ID, were considered if the results for adults with ID were distinguished from the other groups.

Exclusion criteria consist of studies involving cognition disorders/or cognitive dysfunction/(such as dementia, Neurodevelopmental Disorders (NDD), brain injuries, etc.), and children with or without ID. These criteria ensure that the review’s findings are specific to the target population and are not confounded by other groups with different characteristics. Cognitive disorders, such as dementia and brain injuries, often have different underlying causes and manifestations from ID. While some neurodevelopmental disorders (NDDs) can co-occur with ID, they are not the same. NDDs encompass a wide range of conditions, including Autism Spectrum Disorder (ASD), Attention-Deficit/Hyperactivity Disorder (ADHD), and others. These disorders have different symptom profiles and may impact cardiovascular risk differently. Including studies with these participants may introduce heterogeneity and confound the results.

### 2.3. Stage 2: Identifying Relevant Studies

**Figure 1:**
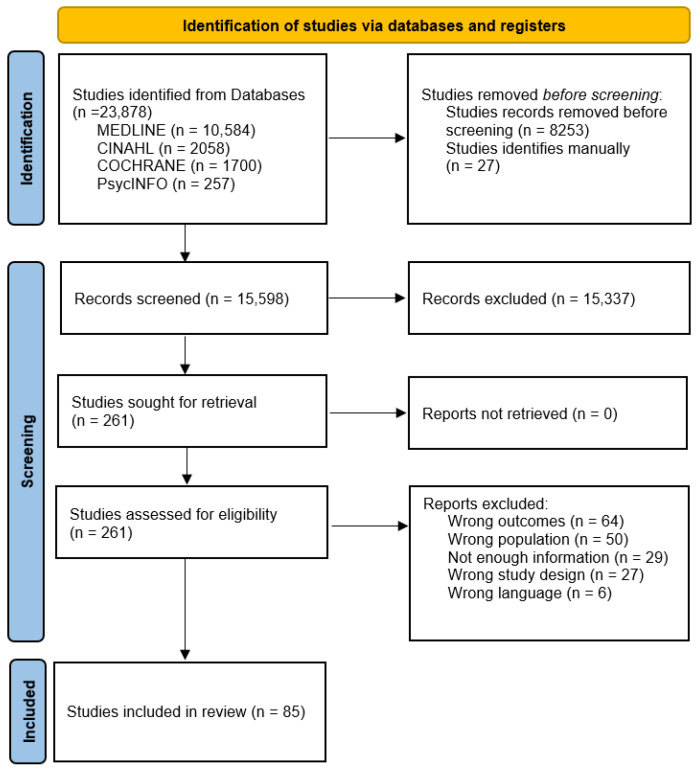
PRISMA flow chart diagram.

A comprehensive search of databases such as MEDLINE, Cinahl, Embase, PsychINFO, Web of Science, Cochrane trials, and Google Scholar was conducted. A combination of MeSH terms and keywords related to intellectual disabilities and cardiovascular risk factors was used. The search strategy for each database was adapted as necessary to align with the specific requirements of each database. Please consult Figure 1.

### 2.4. Study Selection

Our study employed a two-stage screening process, with each record being independently evaluated by two reviewers. The first phase involved screening based on the titles and abstracts of identified studies, using predefined inclusion and exclusion criteria to assess relevance. The second phase involved a detailed full-text review of the selected studies to ensure they were consistent with the inclusion criteria. Throughout this process, we used Covidence, an online tool that supports systematic reviews by facilitating a blind process at all stages [23]. In case of any disagreements or conflicts during the screening process, a third reviewer was consulted.

Here is a list of inclusion/exclusion criteria:

- Population: Studies that include individuals with an intellectual disability aged 18+.
- Interventions/Exposure: Studies reporting on the prevalence of cardiovascular risk factors: hypertension, diabetes mellitus type 2, metabolic syndrome, inactive lifestyle, obesity—overweight—BMI (Body Mass Index), high cholesterol, alcohol and smoking.
- Study characteristics: Quantitative studies—experimental (RCT, quasi-experimental) and observational (cross-sectional, cohort, prevalence, and case–control studies). Studies published after January 2013, and studies published in English and European languages.

We excluded:

- Cognition disorders or/cognitive dysfunction (dementia, neuro-disability disorders and brain injuries)
- Children.

### 2.5. Charting the Data

Data was extracted using the PICO framework [24], which includes information on population, intervention, comparison, and outcomes. The extracted data were systematically organized into a table, detailing authors, year of publication, geographical origin, ethnicity, living conditions, age/sex, study design, research question, type of disability, and outcomes related to cardiovascular risk.

### 2.6. Collating, Summarizing and Reporting the Results

Following the framework of Arksey and O’Malley [22], we presented the narrative account in two ways. First, we described the nature and distribution of the included studies, including elements such as the country of origin, study design, participant characteristics, and setting. Second, we organized the results according to themes that were found to be most relevant to the research questions. Data synthesis was performed using narrative summaries, with data segmented by cardiovascular risk.

### 2.7. Quality Assessment/Risk of Bias

Although quality assessment is not typically a part of the scoping review process, we chose to include it in our methodology. The quality of the studies and potential bias were assessed using the Joanna Briggs Institute (JBI) Critical Appraisal Tools for randomized controlled trials, quasi-experimental studies, observational cohort, and cross-sectional studies [25]. This additional step was taken to strengthen the reliability and validity of the included studies, providing a more robust overview of the available evidence. It also allowed us to evaluate the strength of the conclusions drawn from these studies.

## 3. Results

Following the screening of 15,598 records, 85 studies from diverse global settings were included in our scoping review (PRISMA flow chart, Figure 1). Of these, 48 were cross-sectional studies, 17 were cohort studies, 8 were prevalence studies, 7 were quasi-experimental studies, and 6 were randomized controlled trials (RCTs) (see Supplementary Materials Table S1). A substantial proportion of the studies included originated from the UK (*n* = 14; 16%) and the USA (*n* = 17; 19%).

### 3.1. Population Characteristics

The included studies provided varying levels of demographic information about population characteristics, such as severity of ID, age, sex, and ethnicity.

### 3.2. Intellectual Disability (ID): Types and Severity

The included studies commonly characterize IDs as significant limitations in both intellectual functioning and adaptive behavior. The categorization of ID varies across studies, but often includes specific diagnostic criteria and classification systems or Codes. In 17 (20%) studies, ID was described but not defined [26,27,28,29,30,31,32,33,34,35,36,37,38,39]. The ICD-10 criteria were used to further categorize ID as mild, moderate, severe, and profound [40,41]. ID is also defined using ICD-9 Codes for Intellectual Disability (e.g., 317, 318.0, 318.1, 318.2, or 319), including conditions associated with ID like autism spectrum disorders, DS, Williams Syndrome, Fragile X syndrome, cerebral palsy, and fetal alcohol syndrome [40,41]. Some studies also described ID based on functional assessments, where ID is assessed based on limitations in adaptive behavior, including cognitive and behavioral disturbances, problems with reading, writing, managing money, personal care, telling time, and communication [42]. Liao, Vajdic, Trollor and Reppermund [17] described ID based on preliminary assessments of an individual’s decision-making capacity. The prevalence study by Gawlik, et al. [43] included psychological and educational assessments, such as the Wechsler Intelligence Scale. According to the DSM-IV, moderate ID is demonstrated by IQ scores of 54–35, and severe ID is demonstrated by IQ scores of 34–20. Emerson, et al. [44] used educational qualifications to define ID. Participants were considered to have ID if they scored lower than two standard deviations below the mean on the extracted component and had no educational qualifications, according to the ICD-10.

### 3.3. Demographic Characteristics

Participants ranged in age from 16 to 93 years. Only a small number of studies included participants younger than 18, and even in these cases the majority of the sample consisted of adults or older adults [45,46,47,48,49,50,51]. Across the included studies, 48 studies reported a higher proportion of men, while 28 studies reported a higher proportion of women. A major limitation across studies was the lack of detailed information on ethnicity. When ethnicity was reported, the studies generally indicated a predominant representation of white participants [29,35,41,52,53,54,55,56,57]. Individuals with ID primarily lived alone [32,54], with others in shared housing with staff support [52], or with family or partners [32,54,58,59]. For example, Cocks, Thomson, Thoresen, Parsons and Rosenwax [28] found that 53% lived in a family home, 22% in their own home, and 25% in congregate living. Similarly, Bryant, et al. [60] reported that 29% lived alone, while 71% lived with others, often in shared housing with staff support. Some participants were engaged in employment or day activities, but many were not paid for work. Among those who were employed, working hours were often limited [28,61].

**Figure 2:**
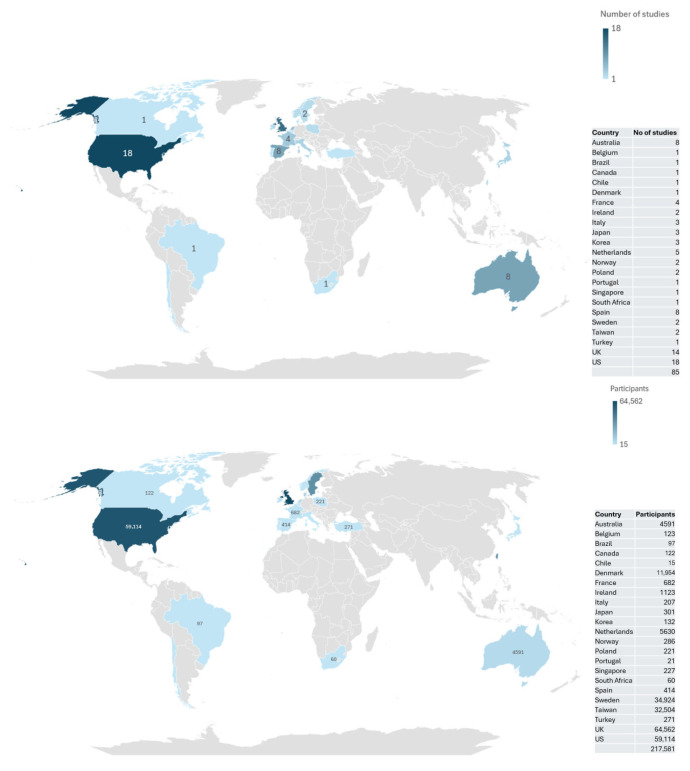
Geographic distribution of included studies (N = 217,581), a Number of studies included, b Number of participants in the included studies.

The geographic distribution of included studies shows that most of the studies were conducted in the US or the UK, enrolling a total of 217,581 participants. Over 80% of participants were from the US, Sweden, and Taiwan (Figure 2).

### 3.4. Cardiovascular Risk Factors

The reported CVD risk factor prevalence included: hypertension, diabetes mellitus type 2, metabolic syndrome, inactive lifestyle, obesity, overweight and higher BMI, high cholesterol (Table 1), alcohol consumption, and smoking (Table 2).

#### 3.4.1. Hypertension

The prevalence of hypertension across 37 studies demonstrated considerable variability, ranging from 0% to 83% (Table 1). Among individuals with DS, the rates were generally low; five studies reported a prevalence of 0%, while the remaining three studies reported prevalence rates ranging from 2.7% to 4.3%. In contrast, individuals with PWS exhibited a higher prevalence of hypertension. Five studies reported rates ranging from 30% to 83%, two studies reported rates of 15.8% and 19.4%, and one study reported a prevalence of 8.7%, although this was observed in an elderly population (>60 years). In studies where ID was not specified, the prevalence varied widely. Six studies reported rates ranging from 2 to 10%, eight studies reported rates between 10% and 30%, and another eight studies reported rates ranging from 35% to 53%.

The considerable variation in prevalence appears to be strongly influenced by the type of ID. Kinnear et al. (2018), reported a 13.6% lower prevalence of hypertension in individuals with DS (4.3%), compared to non-DS individuals (17.9%). Similarly, Nordstrom, Paus, Retterstol and Kolset [49] observed a prevalence of 0% in individuals with DS, 30.0% in individuals with PWS, and 52.4% in individuals with Williams syndrome. Interestingly, among individuals with PWS, growth hormone (GH) therapy during childhood appeared to reduce the otherwise high prevalence of hypertension, which was reported by Kawai, Muroya, Murakami, Ihara, Takahashi, Horikawa and Ogata [76].

The prevalence of hypertension in individuals with ID may increase with age. This was reported by Kawai, Muroya, Murakami, Ihara, Takahashi, Horikawa and Ogata [76] only, who observed an 11.7% lower prevalence of hypertension when adolescents were included in the study population (7.7% in adolescents vs. 19.6% in adults). Similarly, sex did not appear to substantially influence the prevalence of hypertension. Axmon, Ahlström and Höglund [26] and Grugni, Fanolla, Lupi, Longhi, Saezza, Sartorio and Radetti [70] reported only a 1–2% lower prevalence among women compared to men. Additionally, Olsen, Halvorsen, Sondenaa, Langballe, Bautz-Holter, Stensland, Tessem and Anke [50], in a study of 214 participants, found a higher prevalence of hypertension in individuals with severe ID compared to those with mild or moderate ID.

#### 3.4.2. Metabolic Syndrome or Type 2 Diabetes

A total of 21 studies reported on metabolic syndrome and 30 studies reported on type 2 diabetes among individuals with ID. Across the included studies, MetS was more prevalent in individuals with PWS (28.4–56%) than in those with DS (2.7–11%) (Table 1).

Several studies found a low prevalence of type 2 diabetes in DS compared to the general population. This includes a lower odds ratio (OR = 0.4840, *p* < 0.0001) [54], low prevalence across the lifespan [62], but more common in men and women with ID overall [41]. In contrast, Sobey, Judkins, Sundararajan, Phan, Drummond and Srikanth [94] noted a higher prevalence of traditional atherosclerotic risk factors, including type 2 diabetes, particularly in DS individuals older than 18 years.

In Australian adults aged 60 years and above with ID, Hussain, Wark, Janicki, Parmenter and Knox [74] reported significant multimorbidity, with 25.6% diagnosed with diabetes. Individuals with PWS also showed a high prevalence of type 2 diabetes [49,70,83], contributing significantly to their cardiovascular risk profile [101]. Notably, 50% of young adults with PWS were found to have type 2 diabetes in the Korean study by [83].

Few studies reported on medication and treatment. Axmon, Ahlström and Höglund [26] found that individuals with ID in Sweden were 20% more likely to be diagnosed with type 2 diabetes and 26% more likely to be prescribed diabetes medications compared to the general population.

In the ID cohort, 91% of those diagnosed with type 2 diabetes had at least one prescription, and 66% of those with a prescription had a diagnosis [26]. Similarly, O’Brien, McCallion, Carroll, O’Dwyer, Burke and McCarron [5] found that only 7.8% of 551 participants were diagnosed with diabetes; however, they were more likely to receive treatment for hypertension compared to participants with ID without a diabetes diagnosis.

#### 3.4.3. Inactive Lifestyle

Thirty-four studies reported on inactive lifestyles among individuals with ID, which significantly contribute to various CVD factors such as obesity, hypertension, and higher BMI. For example, 60% of participants in one study did not meet national physical activity guidelines [79]. The median time spent sedentary ranged from 411.0 to 542.2 min per day [32]. While Ghosh, Choi, Brown, Motl and Agiovlasitis [69] reported sedentary time ranging from 514 ± 139 min per day, Melville, Mitchell, Stalker, Matthews, McConnachie, Murray, Melling and Mutrie [82] found that 66% of participants spent their time sedentary, walked an average of 4780 steps per day, with 59% having a BMI in the obesity range. In a French study, participants who practiced sports for an average of 5.7 h per week were sedentary for 21.8 h per week [34]. Ghosh, Choi, Brown, Motl and Agiovlasitis [69] reported that American adults with ID spent approximately 8.5 h per day in sedentary behavior, with small differences between sexes, age-groups, and days of the week. In USA, 5.1% of participants had no physical activity, and 41.8% had insufficient physical activity [38]. Harris, McGarty, Hilgenkamp, Mitchell and Melville [32] reported that participants spent an average of 73% of their day in sedentary behavior. Time spent in sedentary behavior was negatively associated with health outcomes [54].

Kim and Yi [77] identified a positive correlation between physical activity and various health markers, including muscular strength and cholesterol levels. Moss and Czyz [82] observed higher objective physical activity levels compared to self-reported levels, indicating potential underreporting of physical activity.

Participants with ID reached significantly higher peak heart rate values during a 6 min walk test compared to non-ID participants [67]. Gawlik, Zwierzchowska and Celebańska [68] reported healthy levels of physical activity in 8% of women and 26% of men, noting a negative correlation between physical activity and BMI. Hsu, Chou, Pan, Ju, Tsai and Pan [73] found that men scored significantly higher than women in abdominal muscular endurance and grip strength tests. Hsieh, Heller, Bershadsky and Taub [41] did not find a significant relationship between adulthood stage and physical activity levels.

#### 3.4.4. Obesity and Body Mass Index

A total of 62 studies examined BMI and obesity, identifying elevated BMI as a significant CVD risk factor. The BMI range across the population spans from approximately 19.5 to 44.1 kg/m^2^, highlighting the variability in body weight and obesity prevalence [29,41,52,54,58,59,60,79,102,106]. (Table 1).

Some studies reported that women with ID are particularly vulnerable to severe morbid obesity and related CVD risk factors compared to males [14,43,68,86,102]. The proportion of obese participants was higher in females than in males (*p* < 0.001) [86]. Specifically, 42% of women and 33% of men were obese, with abdominal obesity noted in 75% of women compared to 47% of men [68]. Over half of the population exhibited excess body weight, with obesity present in 30% of female participants and 19.4% of male participants [43]. Hsieh, Rimmer and Heller [14] noted a marginally significant impact on obesity, with a higher risk of morbid obesity observed in women with ID. Body Fat Percentage ranged from 17% to 32% for males and 17% to 42% for females in an American study by Woods, Knehans, Hoffman, Turner, Arnold, Dionne and Baldwin [102].

#### 3.4.5. High Cholesterol

The prevalence of high cholesterol across 17 studies demonstrated substantial variability, ranging from 2.7% to 82% (Table 1 and Table 2). Among individuals with DS, the rates were generally low; three studies reported prevalence rates ranging from 2.7% to 11.6%, while one study reported a prevalence of 54.8%. In contrast, individuals with PWS exhibited a higher prevalence of high cholesterol. Two of the three studies reported rates of 55% and 82%, respectively, while the remaining study reported a lower prevalence of 10.5%. In studies where ID was not specified, prevalence rates also varied widely, ranging from 8% to 37.2%. Specifically, two studies reported rates ranging from 8% to 10%, four studies reported rates between 10% and 20%, and five studies reported rates ranging from 20% to 37.2%.

The considerable variation in prevalence may be influenced by the type of ID. Hsieh, Murthy, Heller, Rimmer and Yen [42] reported a prevalence of 14.0% in the total sample population; however, among individuals with Dow, the prevalence was 11.6%. Similarly, Nordstrom, Paus, Retterstol and Kolset [49] observed differences across conditions, reporting rates of 54.8% and 55% among DS and PWS, respectively, and a higher prevalence of 61.9% in individuals with Williams syndrome.

#### 3.4.6. Alcohol and Smoking

Alcohol consumption was reported in fewer than 6% of the included studies (six studies), and none identified alcohol as a significant CVD risk factor (Table 2). 

**Table 2:** Prevalence of smoking and alcohol consumption across studies.

Author Year Country	*n*	Type of ID	Age, Mean (Range/±) Men %	Smoking	Alcohol
Bedogni et al. [63] (2020) Italy	45	PWS	26 y 37.8%	4%	
Cocks et al. [28] (2018) Western Australia	328	ID	18–82 y 59%	6.3%	35%
Eisenbaum et al. [65] (2018) USA	13,815	ID	18–75 y 57.1%	Tobacco users: 501 Any tobacco use: 6.3% Dual/poly tobacco use: 10.6% Non-cigarette tobacco use only: 15.8%	
Hsieh et al. [14] (2014) USA	1450	DS	37.1 y 55.2%	Higher smoking in less supervised urban settings	Higher alcohol use in less supervised urban settings
Hsieh et al. [42] (2018) USA	1380	ID	37.05 y 55.4%	3.9% smoked	
Huang et al. [55] (2023) Taiwan	32,444	DS	24.5 y 27.6%	Tobacco: 27.6% Nicotine dependence: 15.5%	0%
Nordstrøm et al. [49] (2016) Norway	72	Williams syndrome, DS, PWS	16–45 y Williams syndrome = 21 PWS = 20 DS = 31	Williams syndrome: 5% PWS: 10% DS: 0%	Weekly: 5% (PWS) 1–3 times a month: 19% (Williams syndrome), 20% (PWS), 16% (DS)
Olsen et al. [50] (2021) Norway	214	DS, mild-severe ID	36.1 y 55.6%	3%	
Real de Ausa et al. [90] (2014) Spain	102	DS	39 y 51%	0%	
Sobey et al. [94] (2015) Australia	4081	DS	39 y 52.8%	3.5%	
Swerts et al. [61] (2017) Belgium	123		45.3 y 48.8%	Smoking: 48% Illicit Substances: 1.6%	45.5%
Tyrer et al. [56] (2019) UK	920	Mild-severe ID	43 y 58%	87.6% never smoked 4.1% ex-regular smokers 8.3% current regular smokers	
Tyrer et al. [57] (2020) UK	1091	ID	33.2 y 58.3%	10.5% were current smokers	
Wee et al. [99] (2014) Singapore	227	Mod-severe ID	46 y 48.5%	2.2%	
Winter et al. [101] (2015) Netherlands	1050	ID, DS, PWS	61.1 y 51.3%	19.6%	Misuse (5 glasses/day): 0.3%
Zwack (2022) [106] Australia	68	Mild-serve ID	31.5 y 64.7%	2.9%	41%

Sixteen studies examined smoking prevalence and its association with CVD risk, showing substantial variation in tobacco use, from as low as 2.2% to as high as 48% among current smokers [99], depending on the study population and how smoking was defined. Several studies reported prevalence rates between 3% and 10% [14,28,42,50,56,57,61,63,65,101].

Among U.S. individuals with ID who used tobacco (*n* = 501), 74% smoked cigarettes exclusively, 11% reported dual or poly-tobacco use, and 16% used other tobacco products. Of those who smoked cigarettes, 80% were daily smokers [66]. Sex differences were evident, with 74.2% of smokers being male compared with 26% female.

Smoking was strongly associated with poor oral health: 87% of participants with gum disease were current smokers, compared with only 4% of those without gum disease [42]. Higher smoking rates were also observed in less supervised urban living environments [14]. Additional studies linked smoking to anxiety and depression. 20% prevalence; ref. [101] and to poorer self-reported health, 67 of the individuals reporting poor health were current smokers [28].

### 3.5. Quality Assessment of the Studies

The quality assessment of the studies included revealed some methodological concerns. Among the six RCTs, three studies did not implement blinding [39,45,88,102]. However, these studies focused on educational interventions or interventions targeting changes in physical activities or healthy eating, which are challenging to blind due to the nature of the interventions. Of the seven quasi-experimental studies, only two included a control group [75,82], limiting the ability to compare outcomes effectively. Additionally, four out of nine cross-sectional studies identified and addressed confounding factors [39,57,101,102], enhancing the validity of their findings. Among the cohort studies, one study [70] had several unclear responses related to exposure, outcome identification, and details regarding follow-up; in addition, confounding factors were not identified. Otherwise, the cohort studies were of good quality. Despite the identified methodological concerns, the overall quality of the studies included was reasonably good.

## 4. Discussion

This review systematically maps the available evidence on cardiovascular risk factors among individuals with intellectual disabilities (ID). The findings highlight several recurring patterns across the included studies and point to a complex risk profile influenced by multiple interacting factors. The evidence indicates that adults with ID frequently present with cardiovascular risk factors such as hypertension, type 2 diabetes mellitus, and obesity. These conditions appear to be shaped by a combination of lifestyle-related factors, medication use, genetic syndromes, particularly DS and PWS, and the severity of the disability. Together, these elements illustrate the diversity of health needs within the ID population and underscore the importance of tailored prevention and management strategies.

A strengthened focus on systematic CVD surveillance and early identification of risk factors in primary health care may support better prevention and management for individuals with ID [108]. Integrating regular CVD-related assessments into routine clinical follow-up could facilitate earlier detection and more tailored care. Implementing such approaches is challenging. For example, in Norway, no national registry data on people with ID exists.

MetS was more prevalent in individuals with PWS (28.4–56%) than in those with DS (2.7–11%) (Table 1). The wide range in the reported prevalence of MetS in individuals with DS aligns with findings by Dass, et al. [108], who similarly reported lower prevalence of MetS among individuals with DS and higher prevalence among those with PWS.

Findings from Wang, Zhang, Svendsen, Li and Li [98] highlight an increased risk of heart failure among individuals with ID, underscoring the need for structured cardiovascular follow-up and tailored interventions. The recent longitudinal study by de Leeuw and colleagues [109] also suggests that future research should further investigate the relationship between CVD incidence and DS, and more broadly deepen the understanding of CVD disease patterns among older adults with ID, particularly because heart failure and stroke appear to be more common in this group.

Emerging evidence also suggests that the severity of ID may be associated with heightened vulnerability to CVD [17,98], emphasizing the importance of integrating physical and mental health services. However, several studies note methodological limitations, including small sample sizes and incomplete reporting of contextual factors such as background and ethnicity. Further research is therefore needed to clarify risk mechanisms and evaluate targeted strategies that support lifestyle adjustments, medication adherence, and the role of caregivers in long-term cardiovascular management.

Across the included studies, there was a general lack of comprehensive reporting on contextual factors such as background and ethnicity. Although some studies, including those by Dunkley, Tyrer, Gray, Bhaumik, Spong, Chudasama, Cooper, Ganghadaran, Davies and Khunti [52] and Tyrer, Ling, Bhaumik, Gangadharan, Khunti, Gray and Dunkley [57] provided more detailed accounts of ethnic distribution, this level of information was inconsistent. For example, two studies reported ethnic composition but offered no further detail on participants’ cultural or socioeconomic backgrounds [29,54]. Such limitations reduce the applicability and transferability of the findings across diverse groups of individuals with ID. More thorough reporting of context, background, and ethnicity is therefore needed in future research to enhance the relevance and interpretability of study results.

Cultural attitudes toward health, diet, and physical activity may influence both the prevalence and recognition of cardiovascular risk factors, contributing to variations in health outcomes [110]. These cultural influences are also relevant for individuals with ID, who may experience elevated behavioral and physiological risk factors shaped by their social context. In some settings, stigma and discrimination toward individuals with disabilities can result in reduced access to healthcare and limited social support, which may further restrict opportunities for preventive care, health education, and physical activity [111]. Cultural norms also shape dietary patterns, activity levels, and healthcare-seeking behaviors, factors central to managing CVD risk [112]. Studies indicate that adults with mild to moderate ID living in community settings may have a substantial burden of CVD risk factors, underscoring the need for culturally informed intervention strategies that address both medical and social determinants of health [113].

Ethnic minority groups with ID, including African American and Hispanic individuals, may face additional barriers to healthcare access and preventive services [114,115]. Studies report higher rates of obesity, hypertension, and diabetes in these groups, contributing to an elevated cardiovascular risk profile. Stigma and discrimination within certain cultural contexts may also lead to reduced community support and increased social isolation, which can negatively influence health behaviors and outcomes [114]

Tailored health programs for individuals with ID who are at risk for CVD should incorporate culturally relevant dietary guidance and physical activity recommendations, as lifestyle modification has been shown to help reduce CVD risk in this population [113]. Given the elevated CVD-related mortality reported among adults with ID [12], it is important to develop primary prevention strategies that are both culturally sensitive and adaptable to diverse community settings. Such approaches can support more proactive health management, including regular screenings and structured follow-up. Evidence from Draheim’s study further illustrates how cultural factors influence health behaviors, dietary patterns, and physical activity, highlighting the need for interventions that acknowledge and respect the cultural backgrounds of individuals with ID [113].

### Strengths and Limitations

While the included studies provided valuable insights into the prevalence of CVD risk factors among individuals with ID, several limitations should be acknowledged. Cultural diversity was insufficiently represented, and many studies offered limited information on ethnicity, cultural norms, or social context. This restricts the ability to understand how cultural factors may influence health behaviors and access to care. Future research should therefore explore these aspects more systematically to clarify their relevance for cardiovascular risk in this population.

Three studies by Zwack [105,106,107] were based on overlapping populations but were included due to their focus on different outcomes, without affecting the overall results.

There is a limited evidence base for intervention studies, underscoring the need for more robust and controlled research designs. Expanding the scope of research to better capture cultural, social, and contextual dimensions is essential for developing interventions that address both physiological risk factors and broader determinants of cardiovascular health. A clearer understanding of how cultural perceptions of disability influence lifestyle choices, social support, and healthcare engagement may contribute to more inclusive and culturally sensitive programs.

As this review encompasses a large body of research and includes a substantial number of studies, it provides a broad overview of the existing evidence base; however, the diversity and variability in study design also reinforce the need for cautious interpretation.

## 5. Conclusions

This scoping review identified 85 studies investigating cardiovascular risk factors among adults with intellectual disability across diverse international settings, more than half of which were cross-sectional. Across the available evidence, a substantial burden of cardiovascular risk factors is consistently reported. Notably, few studies evaluated targeted interventions, underscoring a significant research gap. Addressing this lack of intervention-focused research is essential to guide the development of effective, inclusive, and evidence-informed strategies to improve cardiovascular health and long-term outcomes in this population.

## Figures and Tables

**Table 1:** Prevalence of CV risk factors across studies.

Author Year Country	*n*	Type of ID	Age, Mean (Range/±) Men %	HT (%) Women (w)/ Men (m)	High Cholesterol/Hyperlipidemia *n* (%)	Overweight/Obese Mean (SD) BMI kg/m^2^/%	DM	MetS
Aslam et al. (2022) [3] UK	9917	DS	38 y (28–49) Control 53 (43–61) 45.8%	-	-	BMI 34.3 (8.7) kg/m^2^ CP 33.2 (7.1) kg/m^2^	87.9%	increased IRR of 3.67 (95% CI 2.43–5.55)
Axmon et al. (2017) [26] Sweden	7936	ID/ASD4	≥55 y 54.5%	10.3% w: 10% m: 11%	-	-	20%	-
Baksh, et al. [62] UK	10,204	DS	26 y 47.4%	IRR: 0.26 (0.22–0.32)		IR: 2.02 (1.81–2.25)	IRR: 0.59 (0.52–0.66)	
Bedogni et al. (2020) [63] Italy	45	PWS	26 y (22–30) 38%	56.6%	-	Obese: 69%	20%	56%
Bellicha et al. (2020) [27] France	30	PWS	28.8% (24–33) 0%	-	-	37.2 km/m^2^ 82.3 kg	-	-
Bryant et al. (2018) [60] UK	147	Mild/mod ID	54.4 y (19–83) 30.3%	-	15.8%	32.9 (7.9) kg/m^2^, >40 kg/m^2^ (20.6%).	79%	7.5%
Covian et al. (2023) [58] France	40	DS	28.8 y (18–46) 60%	-	-	w: 25 kg/m^2^ m: 24.7 kg/m^2^	-	-
deAsua et al. (2014) [59] Spain	49	DS	36 ± 11 57% men	0%	-	Obese: 37%	4.5%	11%
deLeeuw et al. (2023) [64] Netherlands	684	borderline/profound ID, 14.9% DS	≥50 y 51.3%	51.3%	19.2%	28.7 vs. 27.3 kg/m^2^	12.8%	31.9%
Dodd et al. (2023) [29] USA	79	DS	45.6%	-	-	Obese: 54.43%	-	-
Dunkley et al. (2017) [52] UK	825	ID mix DS 14%	43 y (SD 14.2) 58%	9%	8%	Overweight: 31% Obese: 68%	73%	5.2%
Eisenbaum et al. (2018) [65] USA	13,815	ID	33 y (18–75) 76.4%	-	-	30.31 kg/m^2^	-	-
Emerson et al. (2016) [44] UK	209	Mild ID		5%	-	Obese: 42%	6%	-
Erickson etl al. (2016) [66] USA	78	Mix	54.8 y (40–79) 53.8%	53.8%	37.2%	-	11.5%	
Farías-Valenzuela et al. (2021) [29] Chile	15	DS	23.1 y 100%	-	-	Body fat pre (25.36 ± 5.60) post (23.01 ± 6.20)	-	-
Fitzpatrick et al. (2020) [53] USA	2342	DS	≥18 y 46.3%	2.7%	2.7% (≥240 mg/dL)	Overweight: 73.43% Obese: 43%	-	2.7%
Fleming et al. (2022) [54] USA	66	DS	38 y (25–55) 48.4%	-	-	Obese: 59%	-	-
Flygare Wallén et al. (2018) [40] Sweden	26,988	DS/ASD3	(0–85)	3.1%	-	Obese: 2.6%	3.1%	-
Front-Farré et al. (2021) [67] Spain	48	Mild-Moderate ID	(60.38 ± 7.5 y) 50%	-	-	29.1 kg/m^2^	-	-
Gawlik et al. (2016) [43] Poland	194	Moderate ID	(20–50) 55.7%	-	-	Overweight: w: 56.9%, m: 55.5% Obese: w: 30%, m: 19.4%	-	-
Gawlik et al. (2018) [68] Poland	27	Moderate ID	28.7 y (21–39) 55.6%	-	-	Overweight: 33% Obese: w: 42%, m: 33%	-	-
Ghosh et al. (2021) [69] USA	52	DS	45 ± 14 y 48%	-	-	Overweight: 26.9% Obese 61.5%	-	-
Grugni et al. (2021) [70] Italy	102	PWS	26.9 y (18.0–50.1) 48%	31.4% w: 30.2% m: 32.7%	-	Overweight: 18.6% Obese: 64.7%	2.94%	28.4%
Harris et al. (2017) [31] UK	50	Mild-profound ID (*n* = 8 DS)	≥18 y 42.1 36%	46.0%	-	40.2 kg/m^2^	3.8%	-
Harris et al. (2018) [32] UK	143	Mild-profound ID	<45 y ≥ 45 43.3%	-	-	Overweight: 16.3% Obese: 48.2%	-	-
Harris et al. (2019) [71] UK	50	Mild-profound ID	40.6 y 30.8%	-	-	Obese: 100%	3.8%	-
Herra-Quintana et al. (2022) [72] Spain	23	DS	29.4 y (21–44) 55%	-	-	Overweight: 52% Obese: 9%	-	-
Hsieh et al. (2014) [14] USA	1450	Borderline, Mild-profound ID	37.1 y (18–86) 55.2%	-	-	Obese: 38.3% Morbid obesity: 7.4%	-	-
Hsieh et al. (2015) [41] USA	4282	DS, ID mild-profound ID	(20–60+) 56.6%	-	-	63.6%	-	-
Hsieh et al. (2018) [42] USA	1381	ID (DS 25.8%)	37.05 y (18–86) 18–44 69.9%	13.0%	13.7%	Obese: 37.2%	6.3%	-
Hsu et al. (2021) [73] Taiwan	60	ID Mild-moderate	39.19 y (19–70) 55%	-	-	Overweight: 35% Obese: 31.82%		
Huang et al. (2023) [55] Taiwan	32,444	DS	27.6% (20 unknown gender)	-	-	57%	11.7%	-
Hussain et al. (2020) [74] Australia	391	PWS	>60 y 62.7%	8.7%	-	-	26%	-
Jacinto, et al. [75] Portugal	21	ID	43.04 ± 11.18 y 52.4%		Cholesterol 174 ± 42.27	28.82 ± 5.53	-	84.28 ± 17.06
Kawai et al. (2023) [76] Japan	425 Adults 162	PWS	Median age 25. (18–48) 14 y (0–48) 50.6%	19.4% (HT and DMT2)	-	17%	40.4%	-
Kim et al. (2018) [77] Korea	17	ID	29.9 y, m: 13, w: 4	-	-	Overweight: 11.76% Obese: 17.65%	-	1.2–13.9 times risk of MetS
Kim et al. (2020) [45] Korea	47	ID	24.6 y 60.5%	-	HDL cholesterol change 190.60 ± 35.52 to 172.60 ± 35.53 mg/dL (*p* < 0.05)	BMI 25.37 ± 5.03 to 25.81 ± 5.07 kg/m^2^ (*p* < 0.05).	-	-
Kinnear et al. (2018) [46] UK	1023	DS 186 nonDS ID 837	43.9 y (16–83) 54.9%	All 15.4% DS 4.3% nonDS ID 17.9%	-	All 40.6% DS 56.5% nonDS ID 37.0%	-	-
Kobayashi et al. (2021) [78] Japan	18	PWS	28 ± 9 years 11 men	83%	82%	mean BMI was 45.1 ± 11.6 kg/m^2^	82%	-
Koritsas et al. (2016) [79] Australia	68	DS, autism	>18 69.1%	-	-	Obese 41%	-	-
Lacroix et al. (2015) [47] France	42	PWD BMI > 30 kg/m^2^	25.5 ± 8.9 (16–60) 38.1%	18	-	All who had BMI >30 kh/m^2^	62%	-
Lazzer et al. (2022) [48] Italy	60	PWS	27± 7 y	-	-	42 ± 12 kg/m^2^	8%	41%
Luchsinger et al. (2023) [80] USA	143	DS	55.7 ± 5.7	12.6% HT medication	-	Overweight 46.9% Obese 27.3%	6.9%	9.8% pre-diabetes
Martínez-Zaragoza et al. (2016) [33] Spain	67	ID mix	34 y (23–50) 59.4%	-	-	Mean BMI: 31.76 kg/m^2^	-	-
Melville et al. (2015) [81] UK	102	Mild-Sevre ID	>18 44.8 y 58.3%	-	-	30.8% 13.5%	-	-
Mikulovic et al. (2014) [34] France	570	ID	(19–59) 59%	-	-	45.6% 17.2%	-	-
Moss & Czyz (2018) [82] South Africa	60	ID	39.6 ± 9.1 (25–62) 50%	-	-	Females higher BMI (31.21 ± 7.79 kg/m^2^) than males (26.82 ± 5.49 kg/m^2^)	-	-
Murthy et al. (2021) [35] USA	1618	ID mix	≥18 y	All 14.0% DS 11.6%	Higher cholesterol among obese than non-obese (19.4% vs. 10.7%)	Obese 19.4%	Increased over time (year 1: 5.7%, year 5: 7.3%)	-
Noh et al. (2022) [83] Korea	68	PWS	24.5 y (19.0–34.0) 57.3%	30.9%	-	-	-	35.3%
Nordstrom et al. (2016) [49] Norway	72	PWS *n* = 20 WS *n* = 21 DS *n* = 31	20–43 y PWS 45% WS 33% DS 39%	PWS 30.0% WS 52.4% DS 0%	PWS 55.0% WS 61.9% DS 54.8%	Obese PWS 80% WS 61.9% DS 64.5%	PWS 15% WS 14.3% DS 0%	PWS 25% WS 14.3% DS 19.4%
O’Brien (2021) [5] Ireland	551	Mixed ID	55.6 (44–92) 43.4%	HT 35.2%	-	-	-	-
Olsen et al. (2021) [50] Norway	214	Mixed ID	36.1 y (16–78) 56% DS, *n* (%) 40 (19) AUD 48 (23) CP 24 (11)	All 6% Mild ID 13% Moderate 2% Severe ID 2%	-	Mild ID 38% obese	-	-
Oppewal et al. (2020) [84] Canada	874 n =122	DS Mix Older	>50 y 61.4 ± 7.8 y 50.3%	19.5%	9.6%	Obese: 2%	-	-
Oreskovic et al. (2020) [85] USA	52	DS	Mean age: 35.1 46%	-	-	Overweight/obese: 75%	-	-
Oviedo, et al. (2019) [86] Spain	37	Mild-serve ID	44 y 57–6%	-	-	28.9 (6.5) Overweight: 42.2% Obese: 31.5%	-	-
Oviedo et al. (2017) [87] Spain	66	Mild-serve ID	>18 Adult 58.3%	-	-	Non-active: 28.6 (6.35), active: 27.4 (5.00)	-	-
Ptomey et al. (2018) [88] USA	150	DS, Autism	36 y	-	-	Obese BMI 37 kg/m^2^	-	-
Pucci, et al. [89] Brazil	97	DS	26.5 (18–56) y 51%	0%	-	Overweight: 40.7% Obese: 25.3%	-	-
Real de Asua, et al. [90] Spain	51	DS	36 ± 11 y 61%	0%	33.3%	Overweight: 19 (37) Obese: 19 (37)	-	10%
Room et al. (2016) [91] Netherlands	193	ID with behavioral problems	37 (18–71) y 81%	5.7%	Cholesterol inhibitors 27.9%	-	-	-
Roy-Vallejo et al. (2020) [92] Spain	26	DS	45 ± 11 y 50%	0%	4%	28.5 ± 2.9	4%	-
Rubenstein et al. (2020) [36] USA	383	ID	>18 y 61.4%	47.6%	-	Overweight w: 26.1%, m: 29% Obese w: 27.3%, m: 22.4%	-	-
Ryan et al. (2021) [37] Ireland	572	Mild-severe ID	(18–65) 42.2%	-	-	Obese w: 69.1% m: 72.2%	-	-
Sarı et al. (2016) [51] Turkey	271	Mild/mod ID	-	SHT w: 3 (3.4), m: 33 (18.0) DHT w: 7(8.0), m: 24 (13.1)	-	Overweight: w: 23 (26.1), m: 53 (29.0) Obese: w: 24 (27.3), m: 41 (22.4) BMI for all: 25.6 ± 5.98	-	-
Schroeder et al. (2020) [38] USA	33,122	ID	31 (SD = 11) y 64%	Among overweight: 31.2%, among obese: 34.0%	-	Overweight: 30.3% Obese: 25.6%	-	-
Shields, et al. [93] Australia	12	DS	18–48 y 80%	-	-	33.7 ± 8.9	-	-
Sobey et al. (2015) [94] Australia	4081	DS	0–89 y 52.8%	2.6%	-	-	3.6%	-
Spanos et al. (2016) [95] UK	28	ID mix	>18 y 36%	25%	21%	-	7%	-
Taggart, et al. [96] UK	31	Mild/mod ID	54.7 y 43.6%	-	-	30.63 (4.97)	-	8%
Tyrer et al. (2019) [56] UK	920	ID	43 y (18–74) 58%	6.7%	-	-	7.3%	-
Tyrer et al. (2020) [57] UK	1091	ID	33.2 y (18–80) 58.3%	-	-	-	7.3%	-
van den Bemd et al. (2023) [97] Netherlands	3356	ID	>18 (55–74) 56.7%	-	-	-	- (wrong numbers in table)	-
Wang, et al. [98] Denmark	11,954	Borderline—Mild ID	62.2%	-	HR: 3.61 per 1000 person-years	-	-	-
Wee et al. (2014) [99] Singapore	227	ID	≥40 y 48.5%	Baseline 15.8% (36/227); post 22.5% (51/227)	Baseline 17.6% (40/227); post 34.8% (79/227)	Obese 10.7%	10.6%	-
Weterings et al. (2020) [100] Netherlands	24	Mild, moderate ID	>18 45.8%	29.2%	20.0%	Overweight/Obese 75%	29.7%	-
Winter et al. (2015) [101] Netherlands	990	ID	>50 y 61.1 y (SD = 8.2) 51.3%	52.8%	23.1%	48.4%	13.8%	44.7%
Woods et al. (2018) [102] USA	19	PWS	34.5 ± 4.3 18–62 y 57.9%	15.8%	10.5%	26.7 Overweight 21.05% Obese 10.53%	15.8%	-
Xu, Choi, Motl and Agiovlasitis [39] USA	58	ID, DS	44 ± 14 y 50%	-	-	34.2 ± 8.4 kg/m^2^	-	-
Yano et al. (2015) [103] Japan	121	Borderline (mild-moderate ID)	57.85%	-	-	24.7 ± 7.7 24.7 ± 5.2 27.3 ± 5.0 †	-	-
Zaal-Schuller et al. (2015) [104] Netherlands	407	Mix	57.98%	11.3% anti HT drugs	Cholesterol inhibitors 6.1%	-	-	-
Zwack, et al. (2021) [105] Australia	39	ID	18–45 y	26%	4.5%	Overweight 77%	-	33.5%
Zwack, et al. (2022) [106] Australia	39	ID	18–45 y	-	-	32.9 ± 8.6	-	-
Zwack, et al. (2023) [107] Australia	35	ID	18–45 y 76.7%	-	-	Overweight 77% 32.8 ± 8.5 kg/m^2^	-	-

† *p* < 0.05 vs. mild intellectual disability.

## Data Availability

No new data were created or analyzed in this study.

## Supplementary Materials

The following supporting information can be downloaded at: https://www.mdpi.com/article/10.3390/epidemiologia7030059/s1, Table S1: Summary table.

## Abbreviations

The following abbreviations are used in this manuscript:

BMI	Body mass index
CVD	Cardiovascular diseases
DMT2	Diabetes Mellitus Type 2
DS	Downs syndrome
HT	Hypertension
ID	Intellectual disability
PWS	Prader–Willi syndrome
